# Molecular and In Vivo Characterization of the High Pathogenicity H7N6 Avian Influenza Virus That Emerged in South African Poultry in 2023

**DOI:** 10.1155/2024/8878789

**Published:** 2024-11-08

**Authors:** Celia Abolnik, Thandeka Precious Phiri, Christine Strydom, Zehaad Ismail, Frances Jordaan, Kaila Wannenburg, Shahn P. R. Bisschop

**Affiliations:** ^1^Department of Production Animal Studies, Faculty of Veterinary Science, University of Pretoria, Onderstepoort, Pretoria 0110, South Africa; ^2^SMT Veterinary Laboratory (Pty) Ltd., Irene, Pretoria 0178, South Africa; ^3^Aspirata-AssureCloud Laboratory (Pty) Ltd., Midrand 1683, South Africa

## Abstract

A high pathogenicity avian influenza (HPAI) subtype H7N6 virus emerged in South African poultry in 2023 and later spread to Mozambique, the first documented emergence of H7 HPAI in the African continent. A total of 6.82 million birds succumbed to the disease or were culled, representing about 20% of the South African egg-laying flock and almost 30% of the broiler breeder flock. The complete genomes of 68 outbreak viruses were sequenced and analyzed, tracing the phylogenetic origins of the ancestral H7N6 virus to a reassortment of various subtypes that circulated in southern African wild birds. Molecular clock analysis determined that the virus emerged in the first week of May 2023, probably in a smallholder chicken flock, before spreading to commercial farms, where the disease was first reported in early June. The multibasic hemagglutinin protein cleavage site (HA_0_) was derived from a nonhomologous recombination event with chicken 28S ribosomal ribonucleic acid (RNA). Few genetic markers associated with an increased risk to humans were present in the translated viral proteins. The intravenous pathogenicity index (IVPI) value of the index case isolate was 1.67, reflecting that 50% of the specific pathogen-free chickens died within 4 days of infection. Surviving birds showing mostly mild clinical signs and recovered by day 10 postinfection. Prior to death, chickens shed the virus primarily through the respiratory route, with lower amounts shed from the cloaca, but in the survivors, the virus was still being shed from the cloaca on day 10. Fomites were the likely source of disease spread between farms, and the amount of H7N6 HPAI virus per gram of feces was calculated at ~383,193 (5.58 log_10_) egg infectious dose 50 (EID_50_) equivalents, chicken feather follicles contained on average 739,712.43 (5.87 log_10_) EID_50_ equivalents, and 20 µg of feather dust contained 14,976.96 (4.175 log_10_) EID_50_ equivalents.

## 1. Introduction

Low pathogenicity avian influenza (LPAI) viruses of all 16 hemagglutinin (H) and nine neuraminidase (N) subtype combinations circulate subclinically in their natural hosts, namely birds of the Anseriformes (ducks, geese, and swans) and Charadriiformes (gulls, terns, and waders) orders [1]. If transmitted to terrestrial poultry, the H5Nx and H7Nx virus subtypes may mutate from the natural LPAI form that causes subclinical infections to a high pathogenicity avian influenza (HPAI) form that causes a highly transmissible and deadly disease with zoonotic potential [1]. Viral virulence is ultimately a multigene trait [2], but the influenza A virus (IAV) hemagglutination (HA) protein is the key determinant, as this protein must be proteolytically cleaved into an active form to initiate infection in the host cell. Random mutations that introduce pairs of basic amino acids (arginine [R] or lysine [K]) adjacent to the HA protein cleavage site (HA_0_) switch the substrate cleavability from host-expressed trypsin-like enzymes that are limited to the epithelial cells of the respiratory and gastrointestinal tracts, to furin or subtilisin-like enzymes that are expressed in a wider range of tissues and organs. Thus, in comparison to the restricted replication sites of an LPAI virus, an HPAI virus is able to spread systemically in the host and cause a more severe infection [3].

LPAI H7Nx has been detected in at least 65 bird species [4], and compared to H5Nx, there have been relatively more emergences of H7Nx HPAI globally. Notable epidemics with severe economic impacts included H7N1 HPAI in Italy in 1999–2000, resulting in the deaths of over 13 million poultry, H7N3 HPAI in Canada in 2004 that caused the destruction of about 17 million birds at a cost of 380 million Canadian dollars, and an H7N3 HPAI outbreak in Mexico in 2012–2013 in which 22.4 million chickens were culled in the attempted control effort [5]. Since the 1990s, reports of humans who became infected with H7 viruses through direct exposure to infected poultry have markedly increased [5]. Normally, H7 viruses only cause mild-to-moderate clinical signs such as conjunctivitis in humans, but a strain of H7N9 LPAI virus that emerged in poultry in China in 2013 and later converted to HPAI infected more than 1500 people with a 39% case fatality rate [3].

Active and passive surveillance studies since the 2000s occasionally detected H7Nx LPAI viruses in wild birds in southern African countries [6–9]. Commercial ostriches are farmed extensively in the semi-desert regions of South Africa's Western and Eastern Cape provinces, and because they have frequent contact with wild birds around food and water sources in the camps and are tested regularly, they inadvertently serve as sentinels for IAVs in the regional wild bird reservoir [8]. H7N1 LPAI virus subtypes were isolated from or detected in ostriches in 1991, 2001, 2012–2013, 2018, and 2020 [8, 10, 11], and H7N7 LPAI was isolated in 2013 [8]. Some of these H7 LPAI strains circulated for several months in flocks, normally causing only clinical signs and mortalities in young ostriches but never converted to HPAI [8]. Farmed ostriches fall within the World Organization for Animal Health (WOAH) definition of poultry, but an H7 subtype virus had never been reported in chickens in the African continent until 2023.

On the 29th of May 2023 (early winter season), a commercial egg producer near Delmas, Victor Khanye Local Municipality in South Africa's Mpumalanga province, logged a sudden increase in daily mortalities. The initial signs were limited to general lethargy and feed refusal, and over the next 3 days, the only clinical signs were a few hens with purple combs and swollen faces. Samples were taken that tested positive for H7Nx IAV. WOAH was notified on 2 June 2023, and the virus was subsequently identified as H7N6 HPAI by sequencing. All the birds on the commercial farm were culled immediately, but an informal farmer with 3000 layers and multiage broiler chickens, located about 500 m from the affected house on the commercial farm, relayed that his bird mortalities had begun increasing in the prior week. A second small-scale farmer in the area was also rumored to have high mortalities and had reportedly sold the carcasses to the public, which the local State Veterinarian and the police were notified to investigate (T. Cilliers, *pers. Comm*). Sporadic cases of H7N6 HPAI in commercial farms in Mpumalanga and the adjacent Gauteng province were reported over the following weeks. In early August 2023, the epizootic exploded, causing the most devastating avian influenza event in South Africa's history. Over 100 outbreaks were reported in commercial farms, with the majority clustered in and around the Gauteng province, affecting egg-layer chickens and broiler breeder flocks. A layer farm in the Morrumbene district in the Inhambane province of neighboring Mozambique, which imports point-of-lay pullets from South Africa, was also affected in October 2023 [12]. In this study, we sequenced the full genomes of all available H7N6 HPAI viruses and performed phylogenetic and BEAST analysis to trace their origin. The virus from the index case was isolated and an intravenous pathogenicity index (IVPI) test was performed in specific pathogen-free (SPF) chickens.

## 2. Materials and Methods

### 2.1. Detection and Diagnosis of H7N6

H7N6 HPAI outbreaks in poultry were diagnosed by accredited South African veterinary laboratories using validated real-time reverse transcription polymerase chain reaction (rRT-PCR)-based methods [9], except that the Eurasian H7 probe sequence [13] was modified because the original version gave inconsistent results in detecting the southern African H7-specific viral ribonucleic acid (RNA) (unpublished laboratory results). The improved H7-specific oligonucleotide probe sequence was: 5′-Reporter-CCR CTG CTY AGT TTG ACY GGG TYK ATC T-Quencher−3′ (modifications underlined). RNA extracts from the swabs or tissues of confirmed H7 clinical cases were forwarded to the University of Pretoria (UP) for genome sequencing. We aimed to include at least one representative virus from each outbreak, where samples were available and viral RNA levels were sufficient. AssureCloud (Pty) Ltd. Laboratory and SMT Veterinary Laboratory (Pty) Ltd. contributed RNA to this study on the authorization of their clients. Cases from backyard and emerging farmers were diagnosed by the Agricultural Research Council—Onderstepoort Veterinary Research laboratory, but the RNA was not made available for sequencing.

### 2.2. Sequencing, Assembly of Full Genomes, Concatenation, BEAST Analysis

Genome amplification RT-PCR was performed on all samples with rRT-PCR cycle threshold (Ct) values < 30 [14]. The RT-PCR amplification products were sequenced with Ion Torrent technology at the Central Analytical Facility of Stellenbosch University using an Ion GeneStudio S5 Prime System (ThermoFisher Scientific, Waltham, MA, USA). Raw data were analyzed at UP, where the methods used for genome assembly, sequence analysis, concatenation, construction of distance matrices, maximum likelihood (ML) phylogenetic trees, and time-scaled maximum clade credibility (MCC) trees were all performed as described elsewhere [9].

### 2.3. Virus Isolation and IVPI Test

A tissue pool from the index case, sampled 31 May 2023 from 47-week-old layer hens on a commercial farm in the Delmas region, was processed, inoculated via the allantoic sac route into SPF eggs, and passaged for virus isolation [15]. The virus isolate, A/chicken/South Africa/SA2310/2023 (H7N6), was titrated according to the method of Reed and Muench [16] and sequenced as above to confirm its purity.

The WOAH-recommended method [15] for the IVPI test was followed using 8-week-old SPF White Leghorn cockerels (*n* = 10) purchased from AviFarms (Pty) Ltd. in Pretoria. The experiment was conducted in a single glove box isolator in the Poultry Biosafety Level 3 facility at UP, with birds provided ad libitum access to water and commercial chicken feed. The second passage stock of isolate A/chicken/South Africa/SA2310/2023 (H7N6), with a titer of 10^7.5^ egg infectious dose 50 (EID_50_)/0.1 ml and a HA titer of 6 Log_2_, was diluted 1:10 in sterile phosphate buffered saline and injected intravenously into each bird. The birds were observed over a 10-day period and scored daily. The surviving birds were humanely euthanized on day 10. All animal procedures were approved by the UP Research and Animal Ethics committees.

### 2.4. Sample Testing

Tracheal swabs, cloacal swabs, and feathers were collected from the fresh carcasses of birds that died or were euthanized in the IVPI test. Swabs were placed into 1 ml of viral transport medium (VTM) [11], and feathers from individual birds were pooled into sterile tubes. Feces from the lower intestines of five chickens that died on day 3 or 4 of the experiment were collected and placed into sterile tubes. 0.2 g of each stool was transferred to a new sterile tube, and 1 ml of VTM was added and thoroughly mixed by vortexing. About 1 mm sections of the feather follicles contained pulp were snipped off using sterilized scissors into 1 ml of VTM with 20 µg/ml proteinase K (Thermo Fisher Scientific, Waltham, USA) and incubated for 1 h at 22°C with occasional vortexing. On day 10, the isolator prefilter was placed into a sterile bio-hazard bag and shaken vigorously to dislodge the feather dust. About 0.1 g volumes of feather dust were resuspended in 1 ml of VTM in sterile tubes and thoroughly vortexed. All samples were stored at 4°C until testing. Total nucleic acids were extracted from 0.2 ml volumes of the swab, feather tip, and feather dust fluids using IndiMag Pathogen kits in an IndiMag 48 instrument (Indical BioSciences, Leipzig, Germany). The relative amounts of IAV-specific RNA were determined by quantitative real-time reverse transcription PCR (qrRT-PCR) using a VetMAX-Gold AIV Detection Kit (ThermoFisher Scientific, Waltham, USA) according to the manufacturer's instructions in a StepOnePlus instrument (ThermoFisher Scientific). A standard curve was generated from a serial dilution of the titrated H7N6 virus included in the qrRT-PCR run. Samples with Ct values < 40 were considered as positive.

## 3. Results and Discussion

### 3.1. Origins of the H7N6 HPAI Virus and the HPAI Multibasic Cleavage Site (MBCS) Cleavage Site

**Figure 1:**
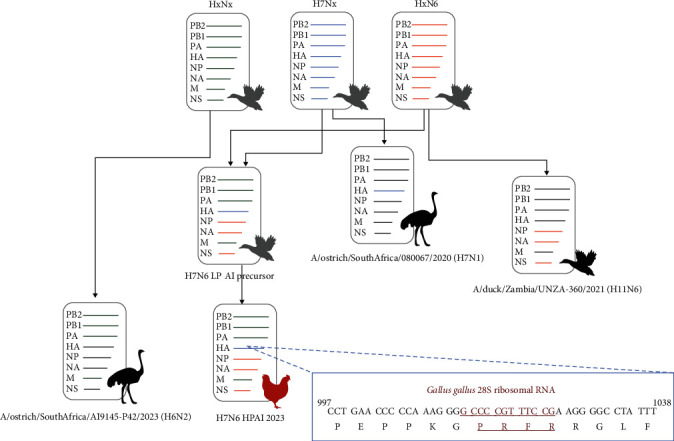
The most RCA of the H7N6 HPAI virus that emerged in South African poultry in 2023 as determined by phylogenetic analysis. Gray bird icons—hypothetical viruses; black and red bird icons—known viruses. Inset—the hemagglutinin protein MBCS site with the insertion in the underlined red script. HPAI, high pathogenicity avian influenza; MBCS, multibasic cleavage site; RCA, recent common ancestors.

The complete genomes of 66 South African H7N6 HPAI viruses and two samples from the outbreak in Mozambique were sequenced, assembled, and analyzed in this study. ML phylogenetic trees for each of the eight genome segments were reconstructed with the closest relative sequences retrieved from the global initiative on sharing all influenza data (GISAID) and National Center for Biotechnology Information (NCBI) databases (Figure S1). The phylogenetic findings are summarized in Figure 1. The H7N6 HPAI virus emerged from an LPAI precursor that shared recent common ancestors (RCA) with strains A/ostrich/South Africa/AI19145-P42/2023 (H6N2) (EPI_ISL_ 19009636) for the polymerase B2 (PB2), polymerase B1 (PB1), polymerase A (PA) and matrix (M) gene-encoding segments, A/duck/Zambia/UNZA−360/2021 (H11N6) (EPI_ISL_18690668) for the nucleocapsid protein (NP), neuraminidase gene (NA) and nonstructural protein (NS)-encoding segments, and A/ostrich/South Africa/080067/2020 (H7N1) (EPI_ISL_12852379) for the HA segment. The latter H7 virus contained the original LPAI sequence at HA_0_ of PEPPKGR*⁣*^*∗*^GLF and had been directly sequenced from the tracheal swab fluids of commercial ostriches during a localized outbreak of H7N1 LPAI in the Mosselbay, Oudtshoorn, Calitzdorp, and Heidelberg regions of the Western Cape province between July and December 2020 in breeders and slaughter-age birds. No clinical signs were observed except for green urine [17]. A/ostrich/South Africa/AI19145-P42/2023 (H6N2), also directly sequenced from tracheal swab fluid, had been sampled from clinically healthy commercial ostriches in the Oudtshoorn region of the Western Cape province during routine surveillance in July 2023.

The H7N6 LPAI precursor virus was not prevalent in southern African wild waterfowl shortly prior to or during the 2023 outbreak, because a national active surveillance project to detect IAV in environmental wild duck feces in 2023 only identified two (0.7%) H7-specific cases, but neither of these were H7N6 strains. For comparison, the H5Nx subtype was detected in 42.2% of all IAV-positive duck fecal swab pools in 2023 [9]. There was no evidence that H7N6 HPAI viruses circulated in wild birds in 2023 [9].

The generation of an MBCS at HA_0_ that switches LPAI to HPAI occurs via one of three mechanisms. The most common event is the extension of stretches of adenine (A) or guanine (G) nucleotides caused by stuttering of the viral RNA-dependent RNA polymerase. The second mechanism is a nonsynonymous mutation of one or more nucleotides in the cleavage site to encode additional R or K residues, and the third is nonhomologous recombination. In nonhomologous recombination, nucleotides from other viral genes, for example, NP or M [18–20] or host sources are incorporated [3, 21]; the South African H7N6 HPAI MBCS at HA_0_ was derived from a nonhomologous recombination event producing a 12- nucleotide insertion that was a 100% match in a BLAST analysis (https://blast.ncbi.nlm.nih.gov/Blast) to the *Gallus gallus* 28S ribosomal RNA gene (KT445934) (Figure 1), similar to the H7N3 HPAI virus in Mexico in 2012 [21].

**Figure 2:**
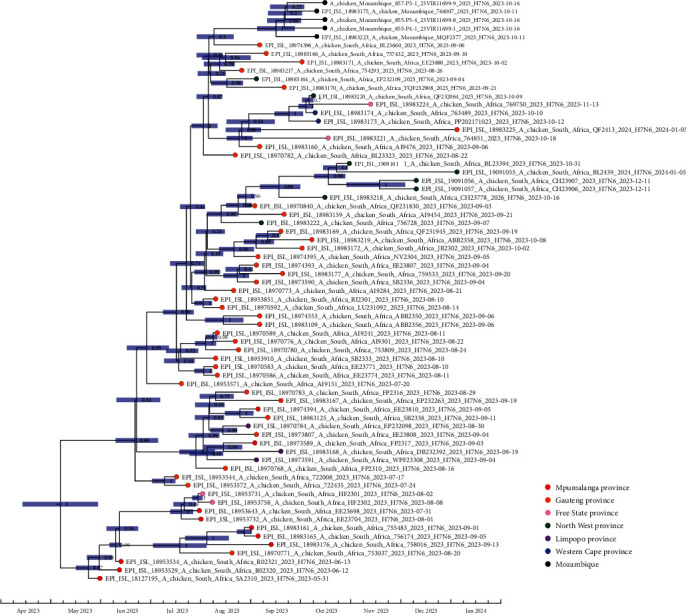
Time-scaled MCC tree of the concatenated genomes of the H7N6 HPAI viruses that caused outbreaks in South Africa and Mozambique's poultry in 2023. The blue bars represent the 95% HPDs of the node, and the posterior probability values are shown. The virus from the rearing farm in the North West province implicated in instances of long-distance spread is highlighted in boldface. HPAI, high pathogenicity avian influenza; HPDs, highest posterior densities; MCC, maximum clade credibility.

In the time-scaled MCC tree produced from the concatenated H7N6 HPAI virus genomes (Figure 2), the ancestral H7N6 virus was dated to the first week of May (mid-April to late May 95% highest posterior density [HPD]), i.e., 1 week prior to the emerging farmers' mortalities and 2 weeks prior to the first clinical signs in the adjacent commercial farm. An MBCS produced in a nonhomologous recombination event can theoretically emerge faster in a single replication cycle, whereas the incorporation of longer stretches of nucleotides via RNA-dependent RNA polymerase slippage occurs in a sequential manner in successive replication cycles [22]. For example, in an experiment where an ostrich-origin H7N1 LPAI virus was passaged in embryonated chicken eggs, the first detectable HPAI sequences with incorporated A's or G's at HA_0_ only appeared after seven passages [23]. Overall, this data indicates that the H7N6 virus was detected within less than a month of HPAI emergence in chickens.

### 3.2. Epidemiological Links in Cases of Long-Distance Virus Spread

**Figure 3:**
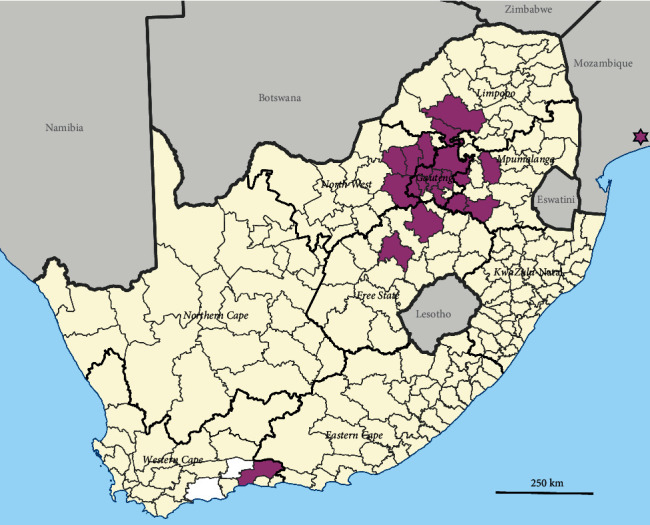
Districts in South Africa and Mozambique (in purple) that were affected by the H7N6 HPAI outbreak in poultry. HPAI, high pathogenicity avian influenza.

The South African outbreaks were centered around a highly poultry-dense region in the north-central part of South Africa, spanning the eastern section of the Mpumalanga province, almost the entire Gauteng province, as well as parts of the North West and Limpopo provinces (Figure 3). The geographic outliers were an outbreak in a layer farm in the Free State province in mid-November, represented in the phylogenetic trees by A/chicken/South Africa/769750/2023 (H7N6), and another cluster in mid-October in a layer farm in George, Western Cape province represented by A/chicken/South Africa/763489/2023 and A/chicken/South Africa/PP202171023/2023. Both layer operations had sourced point-of-lay pullets from the same rearing farm in the North West province. This rearing farm had also reported an outbreak on 9 October 2023, represented in the phylogenetic trees by A/chicken/South Africa/QF232064/2023. The phylogenetic data supports the known epidemiological links for the long-distance transmission of H7N6 between the rearing farm in the North West province and the layer farms in the Free State and Western Cape provinces (Figure S1, Figure 2).

The National Veterinary Authority also reported two “H7N6 HPAI cases” in commercial ostriches from the Oudtshoorn and Hessequa district municipalities in the Western Cape province to the WOAH in 2023. However, the diagnosis on one farm was based solely on the detection of H7-specific ostrich antibodies and an H7-positive real-time RT-PCR result on the other, without sequence confirmation. There were no clear epidemiological links between the layer farm in George and these ostrich farms (L. Roberts, *pers com*.). Based on the lack of any molecular evidence of H7N6 HPAI infection in ostriches and historic trends, in our view, the H7-positive diagnoses in ostriches more likely reflected infections with H7Nx LPAI viruses, as documented on multiple occasions in these regions.

Abnormal mortalities on the layer farm in Mozambique were first recorded on the 25th of September, and within days, the disease had spread to the two other sheds on the farm, ultimately killing 15,000 birds of the 45,000 culled in total [12]. The hens had been sourced in three batches of 17-week-old pullets between 30 August and 30 September 2023 from a single-rearing farm in the North West province of South Africa [12]. Incidentally, this is the same farm that inadvertently supplied the infected pullets to the Free State and Western Cape provinces, but the rearing farm had tested negative for avian influenza before birds were shipped to Mozambique. The RCA of the five Mozambican H7N6 HPAI viruses (two of which were sequenced in this study) was dated to mid-September 2023 (95% HPD first to last week of September; Figure 2), consistent with the timeframe in which the pullets were imported. But surprisingly, unlike the Free State and Western Cape outbreaks, the phylogenetic data does not support the North West province rearing farm, represented by A/chicken/South Africa/QF232064/2023 (Figure 2; Figure S1), as the source of the Mozambique outbreak. From the virus sequences at our disposal, we could not determine the epidemiological source of the Mozambique outbreak, but instead the closest relatives were shared with cases from the Gauteng province.

### 3.3. Zoonotic Potential

The encoded protein sequences of the H7N6 viruses were examined for any mutations known to confer increased infective potential or virulence in mammals. Relatively few markers associated with increased binding to human receptors in the HA, enhanced polymerase activity, or enhanced virulence [2] were detected in any of the translated protein sequences. In the PB2 protein, I292V is associated with increased polymerase activity in mammalian cells and increased virulence in mice [2] and was present in the southern African protein sequences but also in the majority of the Eurasian IAV strains depicted in the matrix protein (MP) phylogenetic tree (Figure S1). K482R is associated with increased polymerase activity in mammalian cell cultures [2], and whereas none of the Eurasian viruses or southern African H7N6 viruses contained this mutation, one H7N6 virus sequenced here, A/chicken/South Africa/PP202171023/2023, had a K482N mutation, the phenotypic effect of which remains to be determined. In the PB1 protein, a D3V mutation was present in the H7N6 HPAI viruses, previously associated with increased polymerase activity and viral replication in avian and mammalian cell lines [2], but this was not unique to the southern African strains as all the Eurasian viruses they were compared to (Figure S1), also contained this mutation. Similarly, the N383D and N409S mutations in the PA protein sequences and the I106M mutation in NS1, all linked to increased viral polymerase activity and/or enhanced replication in avian and mammalian cell lines, were present in all other Eurasian viruses in the phylogenetic trees. However, in the PA protein, the V66I mutation observed in the H7N6 HPAI viruses was not found in other Eurasian viruses or the RCA strain A/ostrich/South Africa/AI9145-P42/2023 (H6N2). A V661 mutation is linked to increased viral polymerase activity and replication in mammalian cell lines and increased virulence in mice, and it possibly emerged here as an adaptation to replication in chickens. In the M1 protein, N30D (increased virulence in mice), I43M (increased virulence in mice, chickens, and ducks), and T215A (increased virulence in mice) in the H7N6 HPAI viruses were also common in the other Eurasian strains. In summary, only eight mutations out of dozens associated with increased virus replication and/or virulence in mammalian cells [2] were detected in the southern African H7N6 HPAI viruses, but these mutations were common in other Eurasian viruses too. Risk to humans of the southern African H7N6 HPAI virus was therefore deemed to be negligible, and no human cases were reported during the 2023 outbreaks.

### 3.4. IVPI

**Figure 4:**
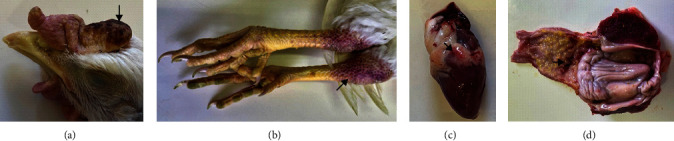
Arrows indicate the macroscopic lesions in chickens infected with H7N6 HPAI virus: (a) necrosis of the comb of a bird that recovered; (b) severe subcutaneous hemorrhage on the hocks; (c) petechial hemorrhages in epicardial fat; and (d) multiple large foci of submucosal hemorrhage at the proventricular–ventricular junction. HPAI, high pathogenicity avian influenza.

Five birds died within 4 days after intravenous challenge with the H7N6 HPAI virus, but the remaining five, some of which showed clinical signs, had recovered completely by day 10 when the experiment ended. The IVPI score was 1.67 (out of a maximum of 3.0), which was slightly above the 1.2 threshold value but still falls within the definition of an HPAI virus [15]. The first clinical signs in the sick birds were depression with ruffled feathers, followed a day later with conjunctivitis, facial edema, and congestion of the combs and/or wattles of six of the birds. Petechial hemorrhages on the hocks were observed in five birds. Birds that developed cutaneous lesions typically died from the challenge. No obvious respiratory or neurological signs were seen. Postmortem examination revealed petechial hemorrhages in the epicardial fat of all the birds, while nine had tracheal hemorrhages and four had proventricular hemorrhages (Figure 4). No macroscopic lesions were observed in the cecal tonsils.

### 3.5. Virus Shedding and Virus in Feathers and Feces

**Figure 5:**
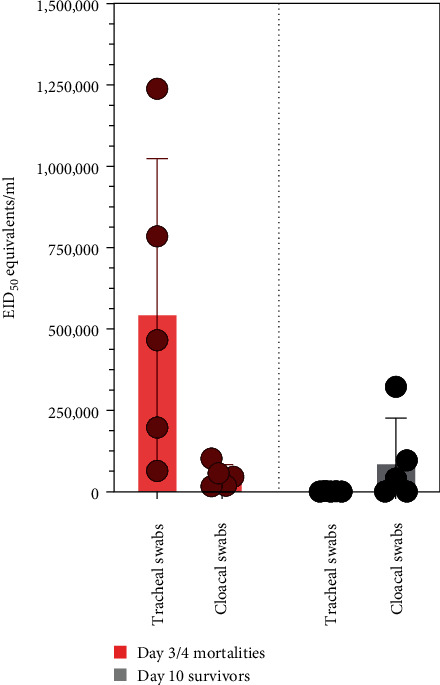
Relative amounts of H7N6 HPAI virus shed from the trachea and cloaca at day 3 or 4 (dead birds) or surviving, recovered birds at day 10. HPAI, high pathogenicity avian influenza.

Swabs were taken from carcasses of birds that died either from infection (day 3 or 4) or from recovered survivors at the termination of the experiment (day 10) to gather information on the primary sites of virus shedding and mode of transmission in an infected flock. The birds that died at day 3 or 4 postinfection shed substantially higher amounts of virus from their respiratory tracts, ranging from 63,532 (4.8 log_10_) EID_50_ to as high as 1,238,046 (6.09 log_10_) EID_50_ equivalents/ml, in comparison to the gastrointestinal tract (Figure 5). Therefore, within an infected house, the virus was likely transmitted primarily via the aerosol route; however, the amount of virus in just 0.2 g of the feces of these early mortalities was calculated to be 76,638 (4.88 log_10_) (±125,768) EID_50_ equivalents of H7N6 HPAI virus, or ~383,193 (5.58 log_10_) EID_50_ equivalents/g.

By day 10, tracheal shedding had almost ceased in the recovered surviving chickens, although one bird was still shedding 40,752 (4.61 log_10_) EID_50_ equivalents/ml in the tracheal swab. Substantially higher levels of virus were shed from the cloaca in three of these recovered survivors compared to the early mortalities, showing that even though chickens appeared clinically healthy, they were still capable of contaminating their environment with large amounts of virus in their feces. Prior studies determined that the bird infectious dose 50 (BID_50_) for chicken-origin HPAI viruses ranged from 16 to 1000 EID_50_ equivalents (median = 398 EID_50_ equivalents) [24], meaning that a single gram of the H7N6 HPAI virus-infected feces could be sufficient to infect ~1000 but possibly as many as 24,000 chickens. The BID_50_ for the H7N6 HPAI virus was not determined here, but we anticipate that it would be in the lower range, judging from field reports of how rapidly the outbreak progressed, whereas rapid aerosol transmission occurred within infected houses, fecal contamination/fomite transmission would have facilitated the virus' transmission between houses and farms over longer distances.

Farmers suspected that strong winds in the region at the time of the outbreaks may have played a role in interfarm transmission of the disease; therefore, we quantified the virus in feathers collected from fresh carcasses and feather dust of the birds in the IVPI test. Feather follicles (~5 per bird) contained on average 739,712 (5.87 log_10_) (±1,968,931) EID_50_ equivalents of H7N6 HPAI virus, and in the feather dust we collected inside the isolator after 10 days, from just 10 birds, 20 µg contained 14,977 (4.18 log_10_) EID_50_ equivalents.

## 4. Conclusions

The South African H7N6 HPAI outbreaks in 2023 represent the first recorded emergence of H7 HPAI on the African continent. The virus first appeared in smallholder chicken producers, speculatively through direct contact with wild birds or the use of untreated surface water inhabited by wild ducks. In practice, smallholder poultry farmers bypass the rigorous surveillance and testing that the large commercial producers are required to implement, and so the circulation of the LPAI progenitor was not detected early on. The outbreak was only diagnosed after HPAI emerged and the first commercial farm became infected. As soon as the smallholder chicken deaths started increasing, sick birds and carcasses were quickly sold by some farmers to recoup their financial loss. No doubt, this aided in the early dissemination of the virus.

Avian influenza is officially a controlled disease in South Africa, and there are clear guidelines for outbreak response that include forward and backward tracing, and increased testing. Infected farms are quarantined and culled, and vaccination was not permitted. The responsibility for disease control rests with the provincial veterinary services but, ultimately, with the National Director of Animal Health. If the existing disease outbreak guidelines had been rigorously followed, the pockets of infection in smallholder farms might have been identified and stamped out early on, and the scale of the H7N6 HPAI epidemic may have been reduced. As the outbreak progressed and increasingly more commercial farms became infected, it became apparent that the virus spread more easily and more rapidly between farms than was experienced with the earlier outbreak of clade 2.3.4.4b H5Nx HPAI in the area (2020–2021), and that biosecurity measures which had effectively slowed the spread of the earlier outbreak were less effective in this outbreak. The situation was further complicated as it emerged that mortality rates in commercial egg layer type birds were lower than those experienced in earlier H5Nx HPAI outbreaks, with mortality rates reported as ranging from 5% to 60% depending on the age and management status of the flocks (Scott Elliott, *pers. comm*). This is consistent with the results of the IVPI test carried out as part of this study, where 50% of challenged birds were able to survive the challenge.

Mortality rates in broiler breeder flocks were reported as much higher (Petrus Engelbrecht, *pers. comm*), necessitating the eradication of the flocks after infection. This difference in clinical outcomes requires further investigation to determine if there is an inherent genetic susceptibility to the infection or whether the difference is perhaps linked to differing management practices in the different industry sectors. Once it became apparent that commercial egg layers were able to survive the challenge quite successfully, farmers became reluctant to cull birds in the absence of compensation from the government. Consequently, the Director of Animal Health made allowance for the quarantining of these affected flocks without enforced culling [25]. These flocks remained alive and were a potential source of further outbreaks. Despite the obvious risks, no restrictions were placed by the national veterinary authority on the movements of live birds, especially across provincial borders.

The outbreak eventually slowed toward the end of 2023, and by the end of January 2024, no further outbreaks in commercial flocks could be confirmed. It was estimated by the South African Poultry Association that a total of 6.82 million birds were dead—either culled or direct deaths from the disease. Of these, 3.97 million were commercial egg layers, and 2.85 million were broiler breeder birds. This represented about 20% of the national egg-laying flock and almost 30% of the national broiler breeder flock. Within Gauteng, the most severely affected province, more than 40% of egg layers had been lost, and more than 80% of broiler breeders were dead [26]. These figures exclude birds affected by the outbreak but not culled.

Very high levels of the virus could be spread in aerosol spread and fomites (feces, feathers, and feather dust), but we still have no clarity on how and why the virus was able to spread so quickly between commercial farms where there were no obvious epidemiological links. A detailed epidemiological and geographical spread analysis is underway to better understand the rapid disease transmission and to allow us to pinpoint critical control areas to mitigate future events.

## Data Availability

The genomic sequences produced in this study were deposited in the GISAID EpiFlu database under accession numbers EPI_ISL_18127195 to EPI-ISL_19091057.

## Supporting Information

Supporting Information Figure S1: Maximum likelihood phylogenetic trees of segment 1 (PB2 gene), segment 2 (PB1 and PB1-F2 genes), segment 3 (PA and PA-X genes), segment 4 (HA gene), segment 5 (NP gene), segment 6 (NA gene), segment 7 (M1 and M2e genes) and segment 8 (NS1 and NEP genes).
