# Supplementary material for: Molecular and In Vivo Characterization of the High Pathogenicity H7N6 Avian Influenza Virus That Emerged in South African Poultry in 2023
Source: Transbound Emerg Dis. 2024 Nov 8;2024:8878789. doi: 10.1155/2024/8878789 (PMC12016866; doi:10.1155/2024/8878789)

**Supplemental Figure 1.**

Maximum likelihood phylogenetic trees of the complete genome sequences of southern African H7N6 high pathogenicity avian influenza viruses (in magenta), and the closest relatives retrieved from public sequence databases. Bootstrap values are indicated.


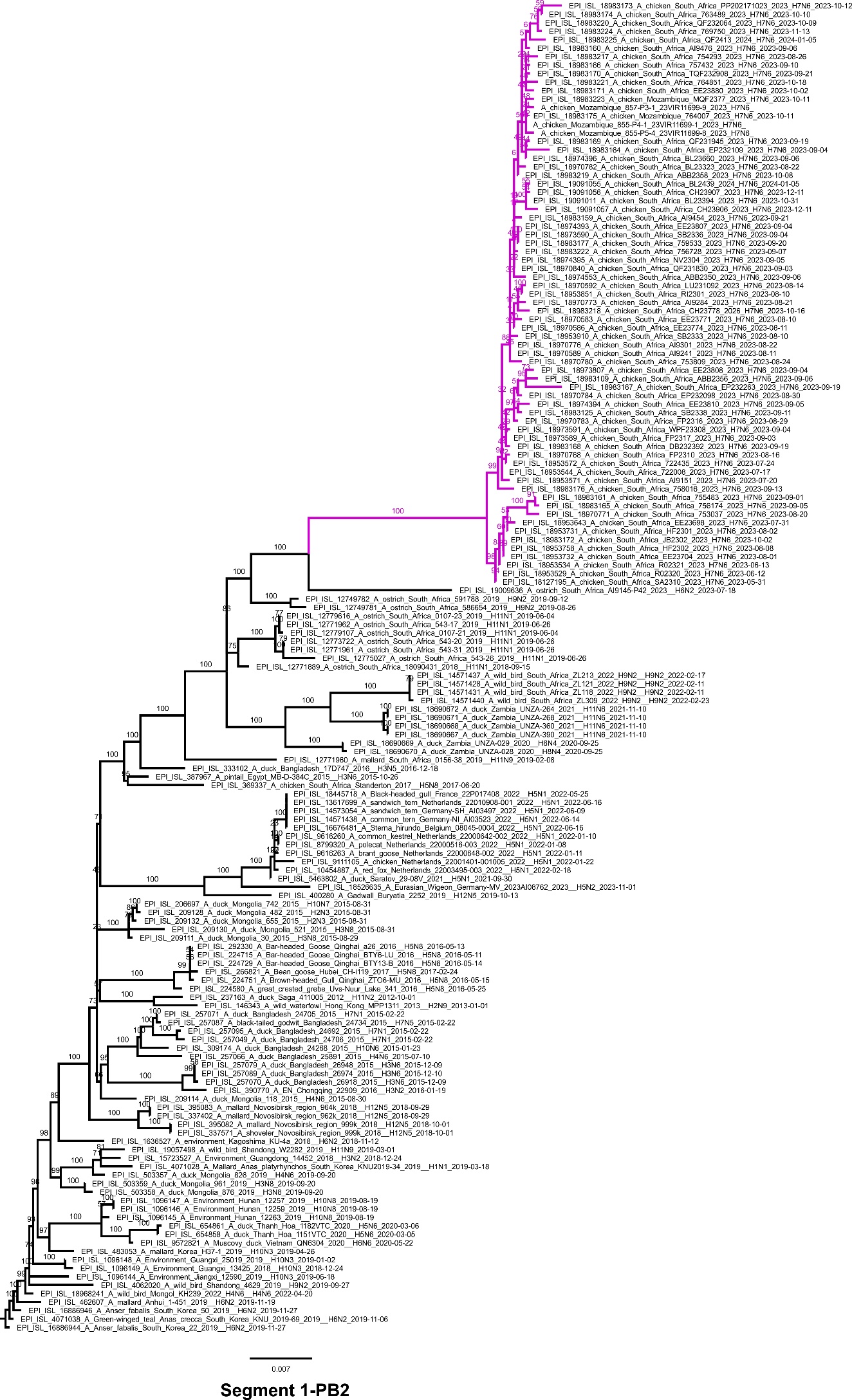


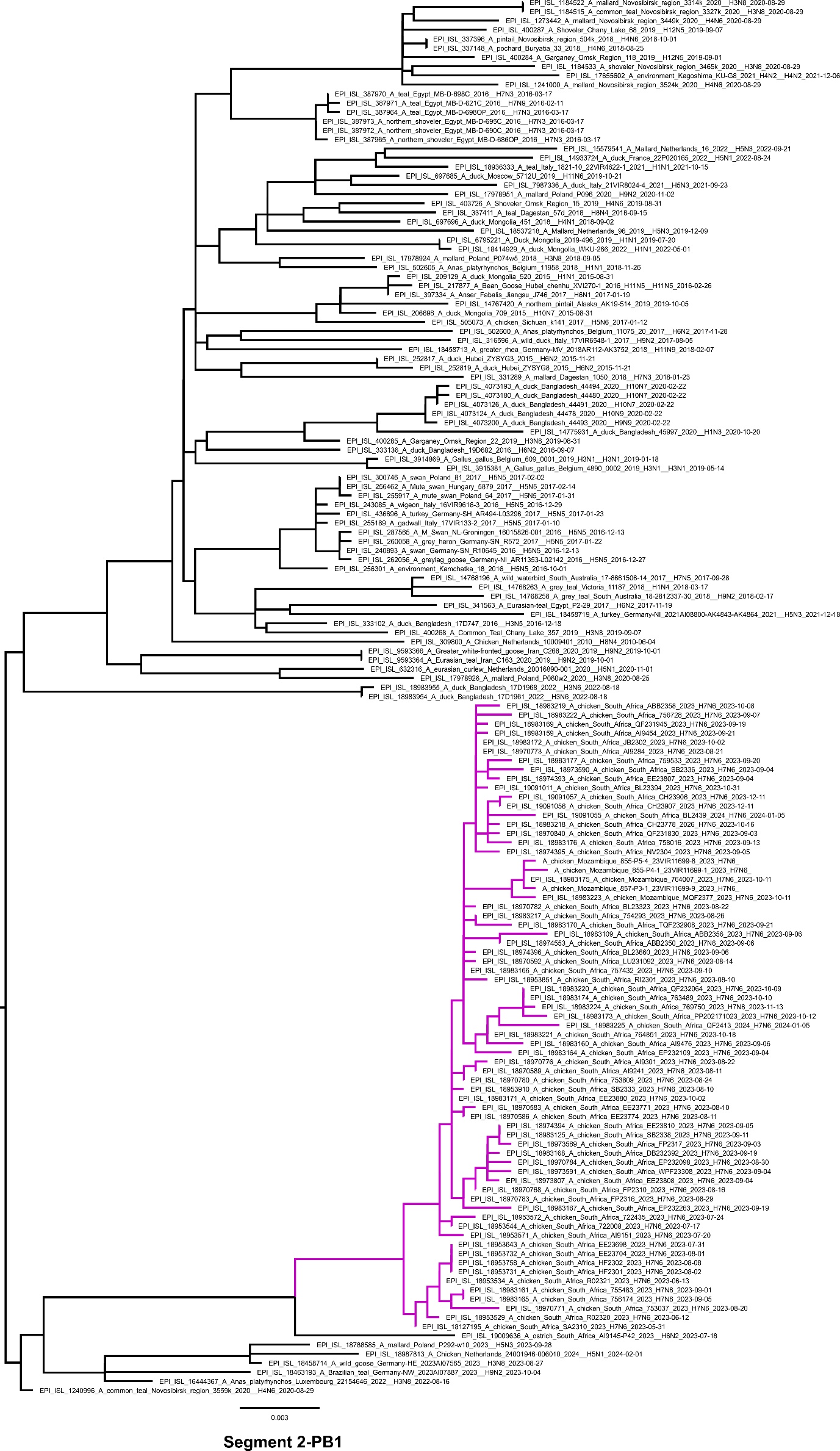


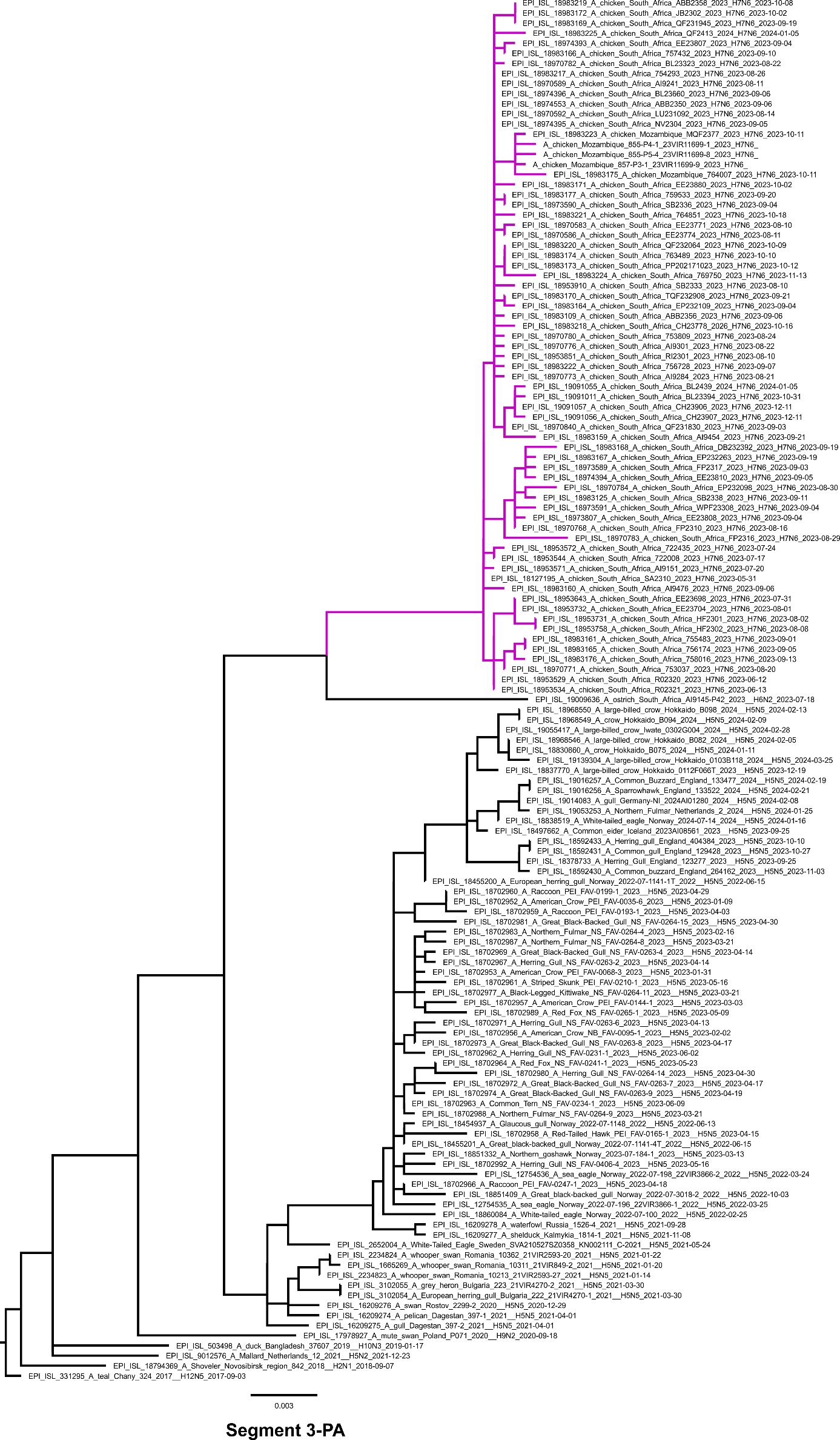


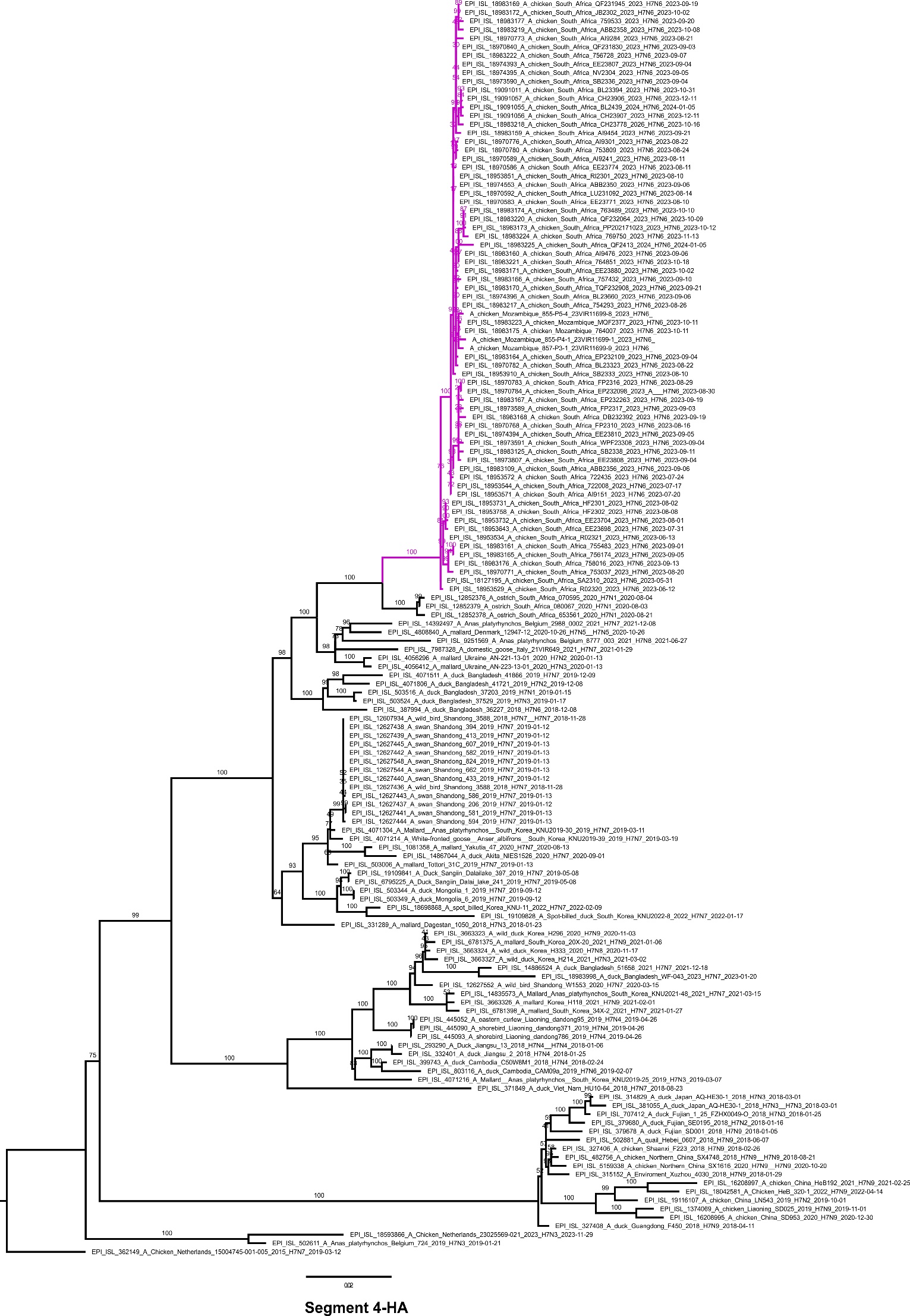


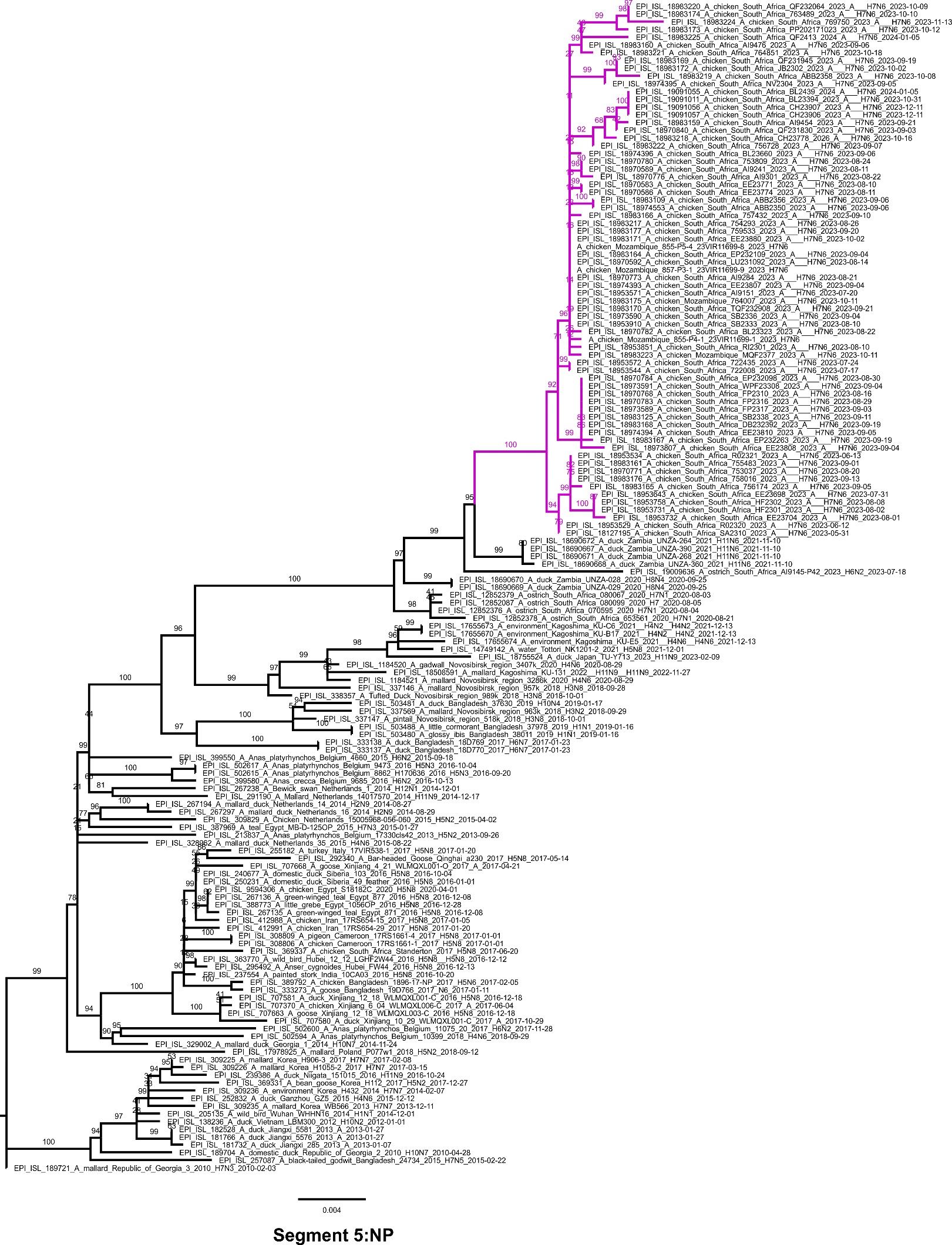


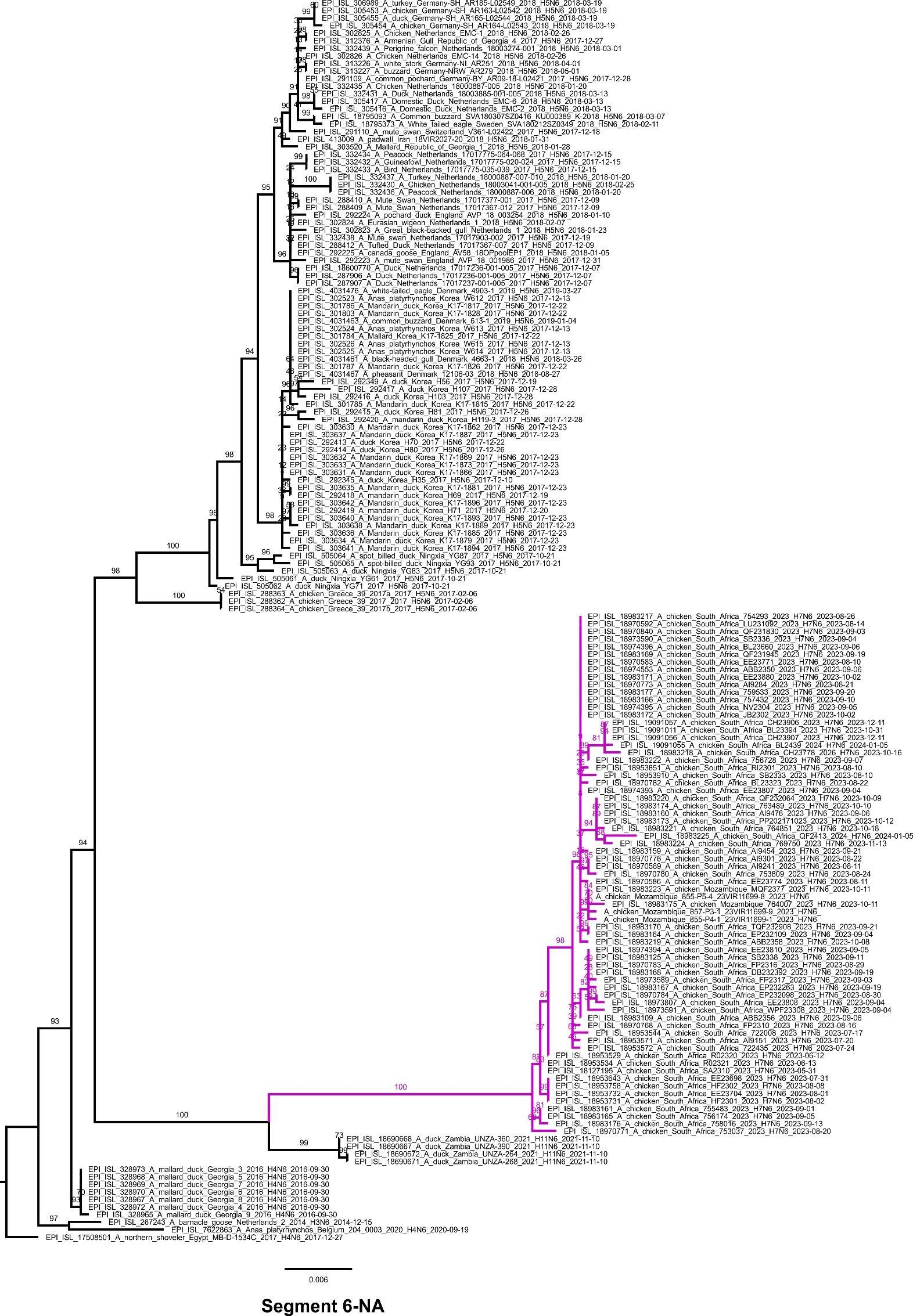


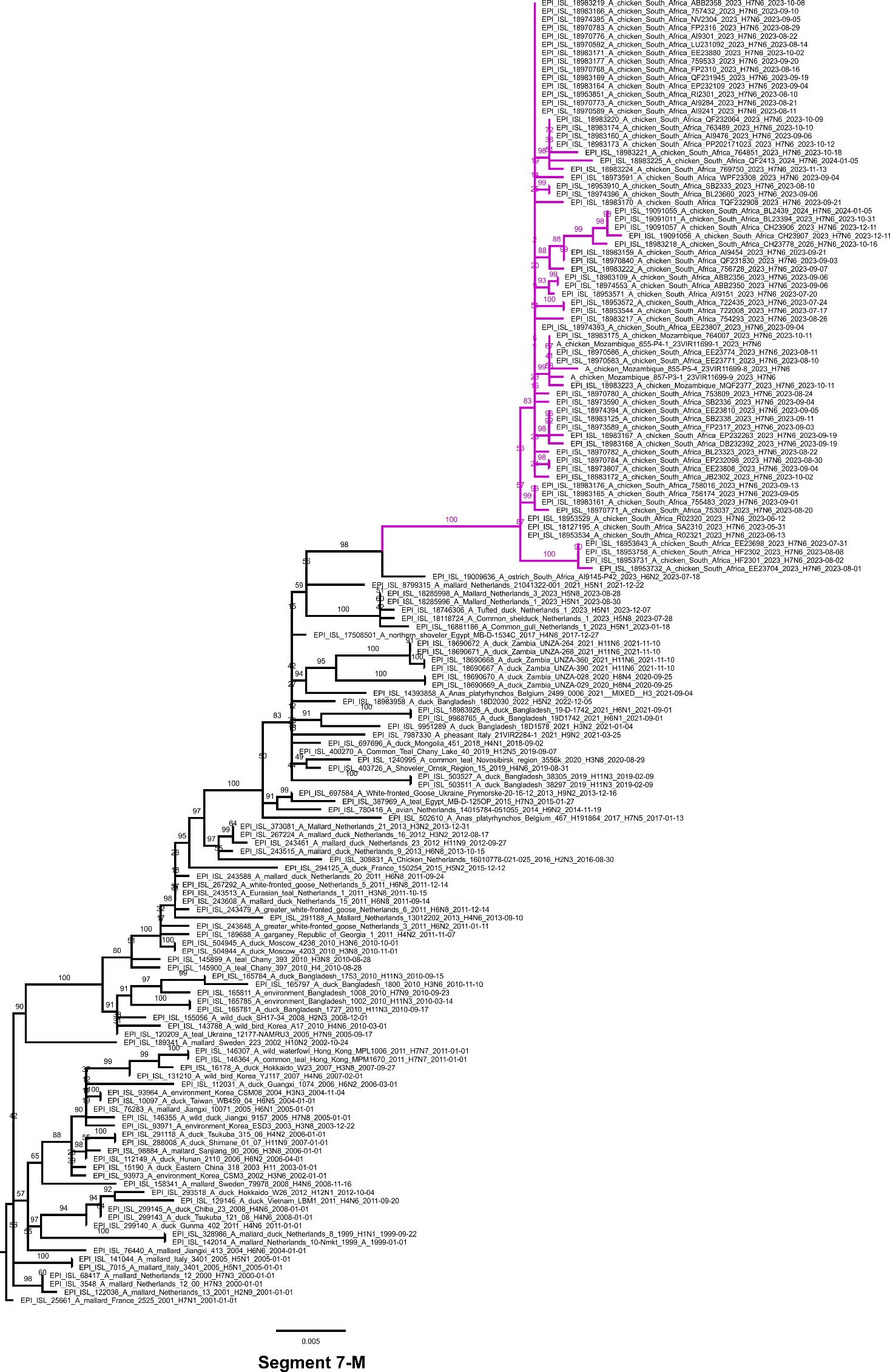


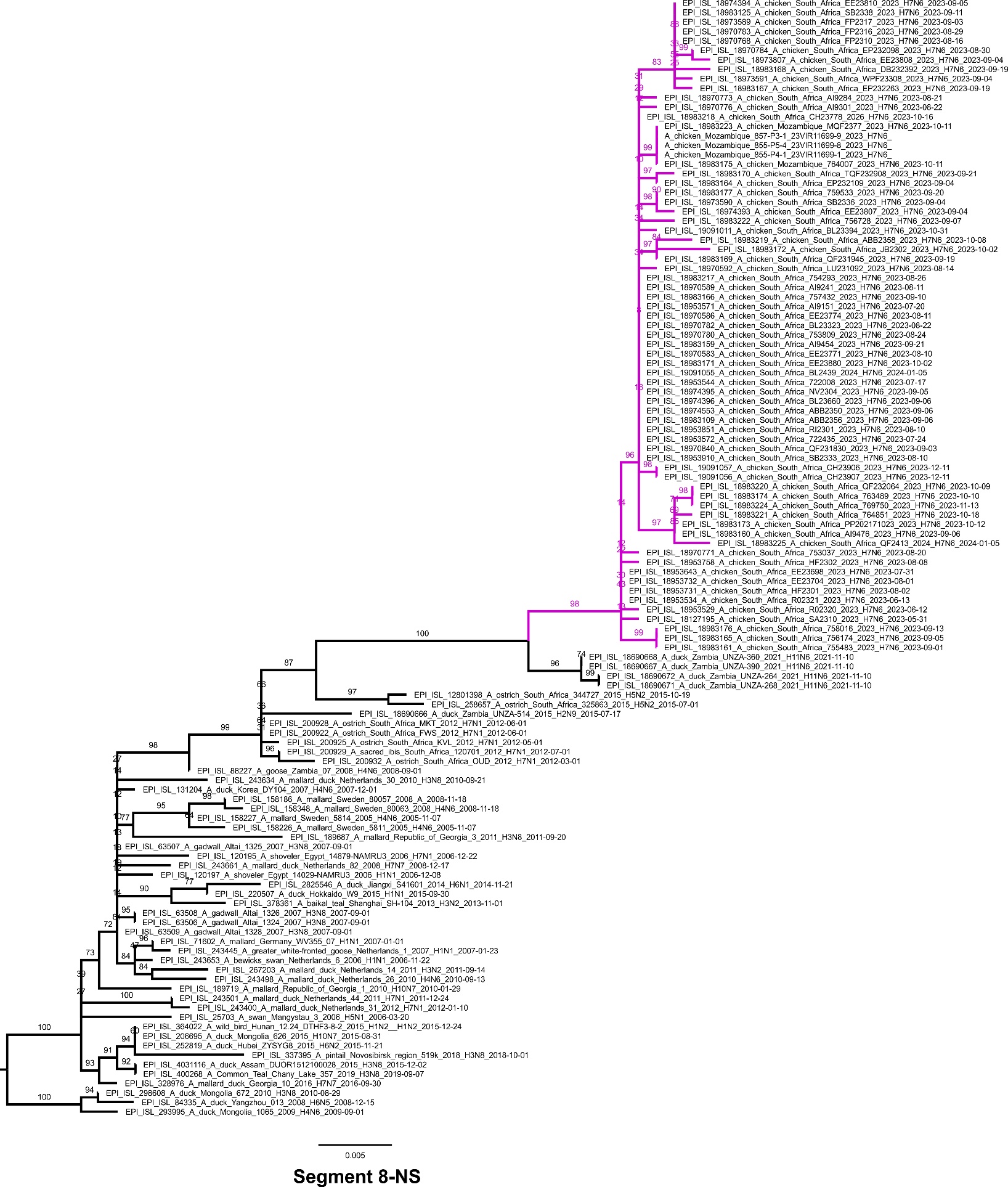

Supplement: Supporting Information — Figure S1: Maximum likelihood phylogenetic trees of segment 1 (PB2 gene), segment 2 (PB1 and PB1-F2 genes), segment 3 (PA and PA-X genes), segment 4 (HA gene), segment 5 (NP gene), segment 6 (NA gene), segment 7 (M1 and M2e genes) and segment 8 (NS1 and NEP genes). [file 8878789.f1.docx]
